# Should I dive or should I float? Behavioural plasticity of *Aedes mariae* pupae under predation threat

**DOI:** 10.1186/s13071-025-06875-z

**Published:** 2025-06-17

**Authors:** Giulia Cordeschi, Valentina Mastrantonio, Roberta Bisconti, Nicole Giardiello, Daniele Canestrelli, Daniele Porretta

**Affiliations:** 1 Department of Environmental Biology, Sapienza University of Rome, Rome, Italy; 2 Department of Ecology and Biology, Tuscia University, Viterbo, Italy

**Keywords:** Phenotypic plasticity, Mosquitoes, Insect behaviour, Pupal stage, Temporary ponds, Water salinity

## Abstract

**Background:**

The pupal stage in holometabolous insects is a critical transition between larval and adult forms, during which feeding ceases and survival depends on stored energy reserves. Mosquito pupae exhibit active diving behaviour in response to threats, which is energetically costly due to their positive buoyancy. Whether pupae are able to adjust diving behaviour according to environmental conditions, balancing predator avoidance and energy expenditure, remains poorly understood. Here, we investigated how water salinity affects the diving behaviour of *Aedes mariae* pupae, a species inhabiting Mediterranean rock pools characterised by highly variable salinity conditions.

**Methods:**

Pupae were maintained and tested in two salinity conditions: low (50%) and high (150%). Diving behaviour was recorded following an automated mechanical stimulus, and we measured: (i) time spent underwater, (ii) pupal activity (i.e. the number of abdominal movements during the immersion and the ratio of movements to time spent underwater) and (iii) the proportion of time spent by a pupa at different depths along the height of the water column (space use).

**Results:**

We found that pupae in high-salinity conditions spent 20.6% less time underwater than those in low salinity. They also performed fewer abdominal movements during dives but showed no significant differences in movements per unit time. Analysis of space use showed that pupae in high salinity spent more time in the upper part of the water column and less time in the middle and lower parts.

**Conclusions:**

*Ae. mariae* pupae modify their diving behaviour in response to different salinity conditions, adopting energy-efficient responses to external stimuli that promote survival in variable habitats. These findings highlight the importance of pupal behavioural flexibility for overall fitness and underscore the need to investigate pupal behavioural plasticity, which remains largely unexplored.

**Graphical abstract:**

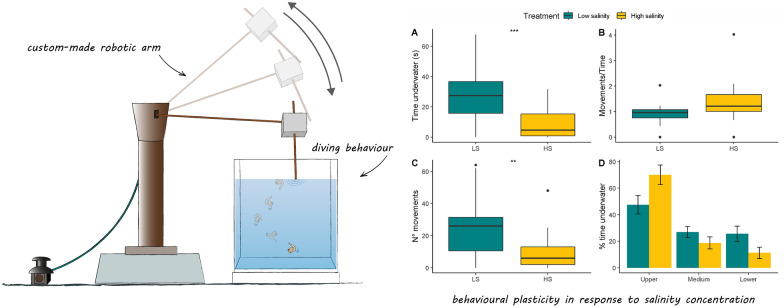

## Background

Holometabolous insects have complex life cycles consisting of multiple stages (eggs, larvae, pupae and adults) [1]. Among these, the pupal stage is a key step between larval and adult phases. However, pupae generally do not feed; they rely on the larval accumulation of resources to survive and transform into adults. Therefore, pupal behaviour is constrained by the need to minimize energy expenditure while still being able to avoid risks (e.g. predators) [2–5]. In many groups, the pupae are immobile, which allows them to conserve resources [6, 7]. However, this is not the case for mosquitoes (Diptera: Culicidae).

Mosquito pupae exhibit two locomotor states: resting at the water surface or diving in response to threatening stimuli, such as a passing shadow or vibrations [8, 9]. Experimental studies have shown that diving behaviour is adaptive, enabling pupae to escape aerial predation and reducing the risk of being washed away from their container habitat by overflowing water [2, 4, 10, 11]. However, this behaviour comes at a cost. Diving is an active, high energy-consuming behaviour for mosquito pupae. Indeed, pupae are positively buoyant due to gas in the ventral air space; therefore, they must counteract buoyancy with continuous body movements to remain submerged, resulting in energy expenditure proportional to the time spent underwater [9, 12]. For example, Lucas and Romoser [9] found that repeated forced diving during the pupal stage significantly increased energy cost, resulting in reduced emergence success and survival in newly emerged adults of *Aedes aegypti* and *Aedes albopictus* compared with individuals from pupae kept at rest. Furthermore, it might also influence adult life history traits. For example, by reducing the adults caloric content [9], frequent diving could negatively affect body size, given their positive correlation [13, 14]. Body size in adult mosquitoes is related to fecundity and fertility, flight ability and mating success [15–20]. Therefore, diving behaviour is critical for population dynamics and persistence.

Mosquitoes can develop under highly variable water conditions, which may affect their diving behaviour [1, 21–24]. To date, studies on the plasticity of diving behaviour have mainly focused on larval stages [4, 23, 25]. For example, in the *Aedes aegypti*, larval diving changes in response to temperature and light, allowing them to avoid heat stress and visual predators [26]. Similarly, *Anopheles gambiae* larvae dive significantly deeper in muddy water columns than in clean ones, balancing the higher energy cost to reach the muddy bottom with improved food acquisition [4]. Whether pupae are capable of optimising their diving behaviour in response to environmental conditions, by managing the trade-off between escape threats and energy conservation, remains unknown.

Here, we investigated the potential effect of the water salinity on the pupal diving behaviour in the sea-rock pool mosquito, *Aedes mariae*. This species develops in rock pools of the supralittoral zone along the western Mediterranean coasts [27, 28]. These habitats are extremely variable, experiencing strong salinity fluctuation over short time periods from freshwater to hyperaline water conditions [29, 30]. Although *Ae. mariae* is not a vector of human diseases, it can contribute to the transmission of avian malaria (*Plasmodium relictum*) [31], and its remarkable adaptation to highly variable salinity environments makes it a valuable model for investigating behavioural responses that may also be relevant to vectors of human diseases developing in saline habitats. Salt concentration affects water density and buoyancy; therefore, we hypothesised that pupae, when exposed to threatening stimuli, would adjust their diving behaviour to conserve energy reserves under different water salinity conditions.

## Methods

Experiments were carried out on *Aedes mariae* pupae obtained from eggs collected from supralittoral rock pools in San Felice Circeo, Italy (41°13′18.77″ N, 13° 4′5.51″ E).

Based on field data collected during the reproductive season of the species, we designed two experimental treatments that differed in salinity. In the first treatment, pupae were maintained at 50% salinity (50 g/l; hereafter referred to as low-salinity condition, LS). In the second treatment, the pupae were exposed to 150% salinity (hereafter, high-salinity condition, HS). Since the eggs were initially kept at 50% salinity, the concentration was gradually increased during the larval stages by 10% per day, reaching 150% at the pupal stage, to prevent salt shock.

After eclosion, second instar larvae were individually placed into plastic trays (12 × 12 × 7 cm) filled with 200 ml of tap water, previously supplemented with aquarium salt (Tetra Marine Seasalt). Individuals were randomly assigned to low- or high-salinity treatment (*N* = 30× treatment). Experiments were conducted in a climate chamber at 26 ± 1 °C, with a 14 h light and 10 h dark regime. Experimental larvae were fed 1 mg of cat food daily.

Diving behaviour was assessed by placing pupae individually in a plastic tray (10 × 10 cm and 9 cm in height) filled with the water of the treatment to which they belonged. Pupae are lighter than water and passively float to the surface when undisturbed [12]. Following a physical disturbance, pupae respond by diving into deeper water and then floating back to the surface to breathe [10]. After allowing the pupa to acclimate for 10 min, we applied a mechanical stimulus by touching the water surface using a custom-made robotic arm to automatically deliver and standardise the stimulus (“Arduino Uno”; http://www.arduino.cc). Individuals were tested randomly with respect to treatment and within 12 h after ecdysis between 8 am and 1 pm. We recorded the pupae diving behaviour at 30 frames/s with an Olympus TG-6 camera placed in front of the experimental tray. Diving tests were carried out in a lightbox with a single LED light (intensity of 1,400 lx) positioned above the experimental tray. Recording started when the mechanical stimulus was delivered and ended when the pupae returned to the surface. Videos were analysed by a single operator (G.C.) blinded with respect to the treatment using the software Behavioural Observation Research Interactive Software (BORIS). The following measures were obtained: (i) the time spent underwater after the stimulus; (ii) pupal activity (i.e. the number of abdominal movements during the immersion and the ratio between movements and time spent underwater); (iii) space use (i.e. the time spent in the upper, middle and lower parts of the water column, 0–3 cm, 3–6 cm and 6–9 cm from the water surface, respectively). We measured pupal body mass to check for its effect on diving behaviour. Body mass was measured by recording pupal weight to the nearest 0.1 mg using a precision balance (Ohaus PR series PR224) within 2 h after pupal ecdysis and prior to diving tests.

Generalised linear models (GLMs) were used to assess the effect of salinity on diving behaviour. We applied GLM with a Gamma distribution and inverse link function for time spent underwater during diving and for the percentage of time spent in the upper, middle and lower parts of the water column. A GLM with a Gaussian distribution and identity link function was used for the number of movements per time spent underwater. To assess the effect of treatments on the number of abdominal contractions during diving behaviour, we applied GLM with a negative binomial distribution. We relied on the Akaike information criterion (AIC) to identify the distribution type and the link function that best improved model fit. We applied GLM with a Gaussian distribution and identity link function for pupal weight and included sex as an independent variable, given the sexual dimorphism of this mosquito species [32]. To test the effect of body mass on diving behaviour, we included it as a covariate in the GLMs. All statistical analyses were performed in R version 4.1.2 (http://www.R-project.org/).

## Results

**Fig. 1:**
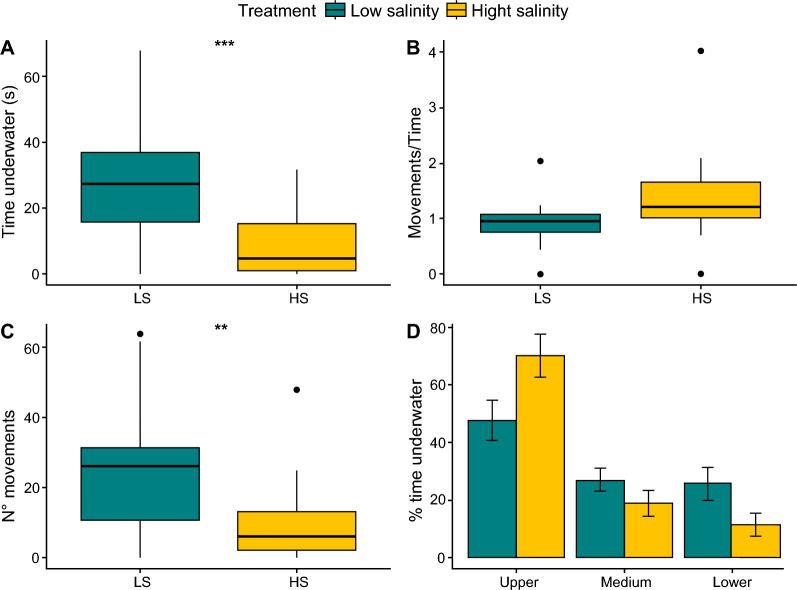
Diving behaviour of pupae. **A**, Time spent underwater in low- (LS; *N* = 20) and high-salinity (HS; *N* = 21) conditions. **B**, Number of abdominal movements. **C**, Ratio between abdominal movements and time spent underwater. **D**, Percentage of time spent in the upper, middle, and lower parts of the water column. Significance levels: ***P < 0.001, **P < 0.01, *P < 0.05, ns: *P* > 0.05. Boxplots show median values (middle line), interquartile range (box) and range values, including some outliers (dots that extend beyond the min and max of the boxplot)

We found a significant effect of water salinity on the diving behaviour of *Aedes mariae* pupae (Fig. 1). The analysis of the time spent underwater showed that pupae from the high-salinity treatment spent 20.6% less time underwater after the stimulus than pupae from the low-salinity treatment (*F*_(1, 37)_ = 19.09, *P* < 0.001; Fig. 1A). The analysis of pupal activity showed no significant effect of salinity conditions on the ratio of movements per time spent underwater (*F*_(1, 37)_ = 4.08, *P* = 0.05; Fig. 1B) but revealed a significant effect on the number of abdominal movements. Indeed, pupae under high-salinity conditions performed fewer abdominal movements than those under low-salinity conditions (*F*_(1, 37)_ = 8.36, *P* = 0.003; Fig. 1C). The analysis of space use showed that pupae in low-salinity conditions spent 47.4 ± 31.0% of the time underwater in the upper part of the water column, 26.9 ± 18.4% in the middle part and 25.5 ± 26.1% in the lower part. Under high-salinity conditions, pupae spent 70.0 ± 32.2% in the upper part, 18.7 ± 19.6% in the middle and 11.2 ± 18.4% in the lower part of the water column (Fig. 1D). When comparing the two salinity conditions, pupae in high salinity spent more time in the upper part than those in the low salinity (*F*_(1,35)_ = 3.48, *P* = 0.07) and less time in the lower (*F*_(1,35)_ = 0.97, *P* = 0.33) and middle parts (*F*_(1,35)_ = 2.75, *P* = 0.10) of the water column, although no significant effect was observed between conditions.

**Fig. 2:**
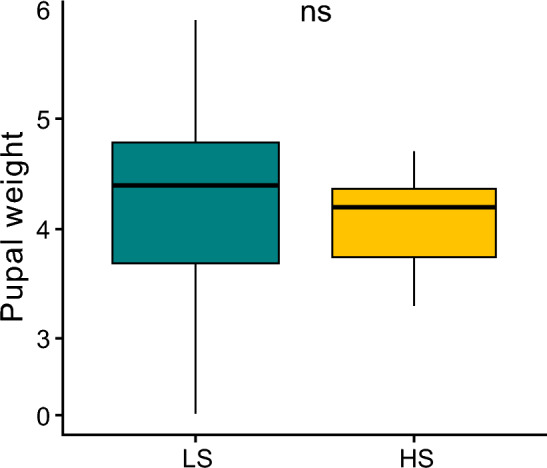
Pupal weight (mg) under low-salinity (LS; *N* = 20) and high-salinity (HS; *N* = 20) conditions. Significance levels: ***P < 0.001, **P < 0.01, *P < 0.05, ns: *P* > 0.05. Boxplots show median values (middle line), interquartile range (box) and range values, including several outliers (dots that extend beyond the min and max of the boxplot)

Pupal body mass did not differ significantly between low- and high-salinity treatments (*F*_(1, 35)_ = 0.79, *P* = 0.37); however, we observed a significant effect of sex (*F*_(1, 34)_ = 19.74, *P* < 0.001; Fig. 2). Neither body mass nor the interaction between body mass and water salinity had a significant effect on the time spent underwater (*F*_(1, 36)_ = 1.24, *P* = 0.27; *F*_(1, 35)_ = 0.55, *P* = 0.45), the ratio of movements per unit time (*F*_(1,36)_ = 0.23, *P* = 0.63; *F*_(1, 35)_ = 1.35, *P* = 0.25) or the number of abdominal movements (*F*_(1,36)_ = 1.06, *P* = 0.30; *F*_(1, 35)_ = 1.04, *P* = 0.30). We did not observe a significant effect of body mass or the interaction between body mass and percentage of time spent underwater on space use (upper: *F*_(1, 34)_ = 0.07, *P* = 0.78, *F*_(1,33)_ = 1.04, *P* = 0.31; middle: *F*_(1, 34)_ = 0.00, *P* = 0.99, *F*_(1, 33)_ = 0.07, *P* = 0.78; lower: *F*_(1, 34)_ = 0.09, *P* = 0.76, *F*_(1, 33)_ = 1.12, *P* = 0.29).

## Discussion and conclusions

Diving is a key component of larval and pupal behaviour in mosquito species. Larval diving behaviour has been extensively studied across different species and environmental conditions [4, 22–26, 33]. Experimental studies have shown that larval diving is affected by a variety of environmental conditions, including predation risk, food availability, heavy rains, water depth, temperature and turbidity [4, 34]. In contrast, pupal diving behaviour has received less attention [35]. Overall, our results demonstrate that *Ae. mariae* pupae adopt a low-cost energy behavioural strategy when responding to external threat stimuli under conditions of high salinity, supporting the hypothesis that diving behaviour is traded off between the need to avoid energy expenditure and escape threats.

This behavioural response under high-salinity conditions might not be unique to *Aedes mariae*. Indeed, approximately 5% of mosquito species live in brackish or saline waters [36], including vectors of human diseases, such as *Anopheles melas*, *An. merus*, and *Aedes taeniorhynchus* which are known to tolerate high salinity [37, 38]. Although pupal behaviour under salinity stress remains poorly documented in these species, the physiological challenges imposed by saline environments are likely to select for energy-conserving behaviours during vulnerable life stages [39]. Moreover, the behavioural strategies observed in *Ae. mariae* may gain further relevance under future climate scenarios. Global warming is expected to expand the extent of saline and brackish water bodies, leading not only to higher densities of salinity-tolerant mosquito vectors but also to the potential adaptation of currently freshwater species to brackish habitats [40]. For instance, both *Aedes aegypti* and *Aedes albopictus* have been reported to oviposit and complete development in brackish water collections [41, 42]. These trends underscore the need to understand the behavioural plasticity of mosquito pupae in varying osmotic environments.

Our findings highlight the importance of pupal behavioural plasticity for mosquito fitness and provide a foundation for exploring how pupae of different mosquito species, including human disease vectors, might respond to environmental stressors.

## Data Availability

The datasets used and/or analysed during the current study are available from the corresponding author on reasonable request.
